# American Medical Women's Association's commitment to advancing menopausal health equity and awareness

**DOI:** 10.3389/frph.2026.1867746

**Published:** 2026-07-03

**Authors:** Meghan Etsey, Rhea Manohar, Aida Mansour, Leah Liszak, Chelsea Oppong, Sarv Raafati, Kiana Wells, Vashti Price, Vaishnavi J. Patel, Teresa Lazar, Ariela L. Marshall, Rosy Thachil, Brianna Clark, Roberta Gebhard

**Affiliations:** 1 St. George University School of Medicine, True Blue, Grenada; 2 George Washington University, Milken School of Public Health, Washington, DC, United States; 3 The University of Texas at Austin Dell Medical School, Department of Population Health, Division of Family Medicine, Austin, TX, United States; 4 Department of Obstetrics and Gynecology, Donald and Barbara Zucker School of Medicine, Hofstra/Northwell Health, Hempstead, NY, United States; 5 University of Minnesota, Division of Hematology, Oncology, and Transplantation, Minneapolis, MN, United States; 6 Division of Cardiology, Elmhurst Hospital Center, Mount Sinai College of Medicine, New York City, NY, United States; 7 Meharry School of Global Health, Nashville, TN, United States; 8 AMWA Gender Equity Task Force Founder, Fellow of AMWA, 2019–2020 President, American Medical Women’s Association, Albany, NY, United States

**Keywords:** hormonal health, hormone changes, menopausal transition, menopause, perimenopause, postmenopause, reproductive aging, women's health

## Abstract

Menopause is a universal yet underrecognized phase of health, with significant implications for physical, mental, and social well-being. Persistent gaps in menopause care, education, and awareness perpetuate gender and socioeconomic inequities, particularly affecting those individuals with comorbid conditions. Menopause-related hormonal fluctuations increase vulnerability to mood instability, exacerbate chronic health conditions, and complicate clinical management. However, insufficient clinician training, lack of standardized screening, limited insurance coverage, and cultural stigma continue to hinder comprehensive and unbiased care. The American Medical Women's Association (AMWA) advocates for menopause equity through systemic strategies, including integrating menopause education into medical training, expanding insurance coverage for menopause-related services, increasing research funding, normalizing menopause in the workplace, launching public health campaigns, and promoting culturally competent care. We believe that advancing menopause equity is essential to improving quality of life, preventing health complications, and ensuring gender-responsive, holistic healthcare throughout the lifespan.

## Introduction

### Defining menopause equity and awareness

Menopause equity refers to the fair recognition, care, support, and inclusion of people experiencing menopause across all social, occupational, and healthcare contexts. It moves beyond simply acknowledging menopause as a biological event to addressing the systemic, cultural, and institutional barriers that shape how it is experienced and managed. Real menopause equity ensures that individuals navigating this phase of life have equitable access to evidence-based care, workplace accommodations, and societal understanding, free from stigma or silence. Additionally, menopause equity acknowledges the historical research gaps in supportive care as well as the impact on the biology of aging, with studies indicating that it is excluded or not fully considered in 99% of aging-related studies (1). With the aging population expected to surpass 2 billion by 2050 and an increasing share being women, it is critical to address menopause equity on a broader scale (2). Menopause awareness, on the other hand, involves expanding both clinical and cultural literacy. This includes, educating clinicians to recognize and effectively manage menopause-related symptoms; training employers and colleagues to create supportive environments; and informing the public that menopause is not a taboo or “women's issue,” but a universal health equity concern affecting families, workplace teams, and communities.

### Why menopause equity is a problem

In the United States, approximately 60 million women are postmenopausal, while an additional nearly 10 million are currently undergoing the menopausal transition (perimenopause) (3, 4). The absence of menopause equity has tangible consequences for women's health, professional trajectories, and workplace safety. Without explicit recognition, support, and education, menopause often becomes an invisible barrier, contributing to workplace discrimination, career stagnation, and adverse health outcomes. Specifically, women may experience symptoms such as fatigue, hot flashes, joint pain, cognitive fatigue, and sleep disturbances (36). These symptoms can increase susceptibility to work-related injuries, including musculoskeletal injuries (e.g., rotator cuff strains), and exacerbate stress-related illnesses, particularly in physically demanding roles or those involving repetitive tasks (5). Without accommodations, women are left to mitigate the exacerbated risk of injury and long-term disabilities, professional consequences of cognitive lapses, or persevere in inflexible workplace conditions alone.

Access to workplace accommodations, such as flexible schedules, modified duties, ergonomic adjustments, temperature control, and rest areas, is crucial for preventing injuries, maintaining productivity, and supporting professional longevity. For those working in high-risk environments, such as hospitals, labs, and construction, workplace accommodations include the development of personal protective equipment (PPE) that minimizes the risks of fainting or overheating associated with hot flashes, a common menopause symptom (6). When these accommodations are unavailable or stigmatized, women may experience higher rates of absenteeism, burnout, and workforce attrition, further reinforcing gender inequities.

Menopause equity also intersects with other aspects of lower social determinants of health. Women living in disadvantaged neighborhoods with unfavorable environmental and social landscapes may experience menopause up to two years earlier compared to those living in favorable neighborhoods (37).

Cultural narratives also shape the experience of menopause. Mainstream media and sitcoms frequently portray menopause through humor and often caricature, exaggerating hot flashes, mood swings, or forgetfulness. While humor can sometimes normalize complex topics, these portrayals often trivialize and belittle real challenges, perpetuating stereotypes that menopause is a punchline rather than a legitimate health concern. Such narratives can discourage women from seeking medical care or requesting workplace accommodations, leaving them vulnerable to both physical injury and professional marginalization. Conversely, respectful and accurate representation, through media, public education, and professional awareness, can help women by validating their experiences, reducing stigma, and fostering environments where accommodations are both available and normalized.

By addressing both structural barriers and cultural misconceptions, menopause equity becomes a community responsibility. Communities and institutions can share responsibility in ensuring that women can continue to contribute fully and safely in the workplace while protecting their health and professional well-being.

### How menopause inequity impacts health outcomes

Menopause-related inequities significantly impact women's health outcomes due to hormonal changes, leading to increased vulnerability to physical and mental health challenges, decreased quality of life, and heightened risk for serious medical conditions. Notably, the perimenopausal and menopause transition phases affect a myriad of health conditions, including cardiovascular disease, osteoporosis, genitourinary symptoms, metabolic changes, and neurological and mood-related disorders (7–9).

As the body adjusts to altered hormone levels, such as decreased estrogen levels, women are at a higher risk for the downstream effects of hormone-modulated pathways. Women with vasomotor symptoms, particularly those younger than 60, may experience increased cardiovascular and metabolic risks such as decreased vascular signaling, insulin resistance, and increased cholesterol (10). Additionally, decreasing estrogen levels accelerate bone loss, leading to heightened risk for osteoporosis and fractures, as well as genitourinary symptoms causing vaginal dryness, urinary urgency, and increased urinary tract infection risk (11, 38). These factors coincide with this transition being a high-risk period for neurological and psychosomatic changes in mood, sleep patterns, cognitive shifts, and other symptoms. Although the overall prevalence of mood disorders may not differ substantially by sex, menopause-related hormonal changes can exacerbate mood and cognitive changes, trigger first-onset symptoms, or worsen preexisting conditions in vulnerable women (12, 13). Notably, heightened anxiety, depressive symptoms, and subclinical mood instability risks are exacerbated in women with prior reproductive-related mood or health events, such as premenstrual or postpartum episodes (14–16).

Diagnosis and management of menopausal symptoms are complicated by overlapping conditions, requiring careful evaluation of reproductive history, symptom chronology, and psychosocial context (13). Hormone therapy remains the most effective treatment for vasomotor symptoms, but individualized strategies are essential to address comorbidities, mood and sleep disturbances, and bone and cardiovascular health (13, 17, 18). Shared decision-making and multidisciplinary care, alongside increased research investment, are critical to optimize outcomes and reduce inequities (1, 15). Nonhormonal and off-label treatments—including gabapentin, clonidine, oxybutynin, higher-dose SSRIs/SNRIs, pregabalin, and suvorexant—are sometimes used when standard therapies are contraindicated or declined, but evidence for their efficacy is limited and adverse effects are common (19–21). Complementary approaches such as black cohosh, soy isoflavones, ginseng, probiotics, acupuncture, exercise, and mindfulness may provide general wellness or stress benefits but have inconsistent or weak data for vasomotor symptom relief (22, 23). Clinicians must balance evidence with patient-specific factors, considering safety, tolerability, and preference, to guide individualized, patient-centered care.

### Causes of gaps in menopause care and awareness

Significant gaps in menopause care and awareness persist, contributing to worsened health outcomes for many women. Key causes include insufficient clinician education on menopause, lack of standardized screening and staging protocols, under-recognition of high-risk subgroups, and persistent gender bias in health messaging and research priorities (13, 15, 24). Patients and clinicians often receive inconsistent or unclear information regarding menopause-related physical and mental health risks, which can lead to underdiagnosis or misattribution of symptoms, delayed intervention, and suboptimal management (13, 24, 39). The absence of prospective studies and standardized monitoring protocols for symptom changes during the menopause transition further compounds this issue, resulting in missed opportunities for early support and personalized care (15, 24).

Evidence-based guidance on interventions, including menopause hormone therapy (MHT), lifestyle modifications, and mental health support, remains limited, leaving many women without clear options for symptom management (13, 17, 18). These gaps are compounded by reports of inadequate patient education and support, while persistent stereotypes regarding menopause and aging further reinforce barriers to care (24). Several important but lesser-known considerations regarding hormone therapy in postmenopausal women warrant attention. The “timing hypothesis” proposes that initiating MHT early in menopause may confer cardiovascular protection; however, current evidence does not confirm this benefit (25). Women with premature menopause (before age 40) represent a critical evidence gap, as they were largely excluded from the Women's Health Initiative (WHI), and current guidelines recommend continuation of hormone therapy until the average age of menopause in these cases (26). Additionally, contemporary formulations, including low-dose transdermal estradiol and micronized progesterone, may have improved safety profiles, but no large, placebo-controlled randomized trials have adequately assessed long-term benefits and risks among younger postmenopausal women with vasomotor symptoms (27). These knowledge gaps underscore the importance of individualized, evidence-informed care and shared decision-making in managing menopause symptoms.

Addressing these gaps requires multifaceted strategies, including improved clinician education, implementation of standardized menopause staging in routine care, targeted screening for high-risk populations, and development of updated gender-sensitive research funding for evidence-based health messaging and intervention protocols (13, 15, 24).

### Precedent for menopausal equity advocacy

Across the United States, momentum is building toward greater recognition of menopause as a workplace and public-health equity issue. In a landmark move, Rhode Island became the first state to amend its employment law to explicitly include menopause-related conditions and require reasonable workplace accommodations (28). This legal precedent establishes a critical foundation for addressing menopause through a lens of gender equity, workplace safety, and employee rights on a broader scale.

Following Rhode Island's lead, several states, including New York, New Jersey, and Massachusetts, have introduced or are considering legislation to improve menopause care, provider education, insurance coverage, and workplace protections. On a national scale, several bills, including the “Menopause Research and Equity Act of 2023,” “Servicewomen and Veterans Menopause Research Act,” and “Improving Menopause Care Act of 2025” have aimed to address menopause equity through increased research and care. While these initiatives remain at various stages of development, their growing presence in state and national legislatures signals increasing momentum toward integrating menopause into broader conversations about health equity and labor protections. However, it is essential that legislative efforts endorse evidence-based strategies, as some proposed bills are championed by stakeholders with conflicts of interest who promote supplements or non-consensus treatments lacking rigorous clinical support. Ensuring that policies are informed by high-quality evidence can safeguard patient safety, support equitable access to effective interventions, and prevent the formalization of practices that may be commercially motivated rather than clinically justified.

In November 2025, the Food and Drug Administration (FDA) moved to remove the “black box” warning from estrogen-containing products, which limited the use of and increased hesitance in MHT use for evidence based menopause care. The side effects of MHT remain common fears by patients, leading to avoidance of the therapy, reinforced previously by the warning even when medically advised (29). This removal highlights a key step towards decreased stigmatization of prescribing estrogen-containing products and empowers clinicians to educate patients and include MHT in individualized menopause symptom management protocols when advised (30, 31). Updated management guidelines, clinician and patient education, and longitudinal and treatment-focused research studies can support women in achieving more equitable and sustainable menopause care and support plans.

The private sector is likewise beginning to respond. CVS Health, a Fortune 10 company, publicly earned a “Menopause Friendly” accreditation, publicly affirming its commitment to supporting employees through formalized benefits, education programs, peer-support networks, and clinical navigation resources (32). Similarly, Alphabet/Google has incorporated menopause support into its benefits framework, offering employees access to flexible leave, health coverage, and workplace accommodations tailored to their needs [Google Careers (33)]. These examples highlight a growing awareness among major employers that menopause equity is not only a health issue but a key factor in workforce retention, productivity, and inclusion.

Comprehensive menopause policies typically include clear guidance on reasonable accommodations, such as temperature control, flexible hours, remote work options, and temporary duty adjustments, along with leave provisions, access to evidence-based treatment (including hormone therapy where appropriate), and structured care pathways. Leading frameworks also emphasise manager training, stigma reduction, confidentiality protections, and data collection to measure impact and equity outcomes [Equality and Human Rights Commission [EHRC], 2024 (34)].

Evidence from international best practices and expert recommendations consistently demonstrates that such measures reduce absenteeism, improve retention, and foster more equitable workplaces. As more states and corporations adopt policies that recognise menopause as a legitimate and supported phase of working life, they lay the groundwork for a broader cultural shift, one that treats menopause equity and education as essential components of gender parity in medicine and beyond.

## Strategies for improvement

Based on the above, AMWA advocates for the following as potential strategies to improve menopause health equity:

1. **Integrate Evidence Based Menopause Education into Medical Training:** Ensure clinicians of all backgrounds, both in training and practicing, receive comprehensive training on menopause, its mental and physical health impacts, and sex-specific care considerations.
2. **Expand Insurance Coverage for Menopause-Related Care:** Include menopause-related preventive screenings including, but not limited to bone density scans, lipid profiles, gynecological exams, and hormone levels, hormone therapy, mental health support, and other menopause-related services under all insurance plans.
3. **Update Evidence Based Menopause Care and Support Protocols:** Highlight FDA-approved and evidence-based menopause treatment protocols, including the use of MHT, in physician education and training materials.
4. **Increase Funding for Menopause Research:** Prioritize studies on menopause, including its intersection with chronic diseases, comorbid diagnoses, and reproductive transitions.
5. **Normalize Menopause in the Workplace:** Implement workplace policies and support systems that acknowledge menopause as a natural life stage and reduce stigma (i.e., flexible schedules, modified duties, ergonomic adjustments, temperature control, rest spaces, and modified PPE).
6. **Launch National Public Health Campaigns on Menopause Awareness:** Educate the public and healthcare professionals about menopause symptoms, health risks, and management strategies led by national public health agencies in partnership with medical and nursing councils, professional associations, employers, educational institutions, community organizations, and media partners to ensure broad, inclusive, and evidence-based outreach.
7. **Promote Inclusive and Culturally Competent Care:** Ensure menopause care addresses diverse populations, including variations in cultural beliefs, access to care, and intersectional health disparities.

## Conclusion

Menopause represents a universal yet deeply individualized health transition that has too often been overlooked in clinical practice, medical research, and health policy. The lack of comprehensive coverage, education, and institutional support for menopause care perpetuates inequities that disproportionately affect women and gender-diverse individuals. Addressing this gap is not only a matter of medical necessity but also of social justice and gender equity.

The American Medical Women's Association calls for a systemic commitment to advancing menopause equity through inclusive research, provider education, equitable healthcare coverage, inclusive workplaces, and culturally responsive care. By integrating menopause into the broader framework of women's and gender health policy, we can begin to dismantle the stigma, misinformation, and inaccessibility that have long hindered progress. Notably, proposed policy and workplace strategies require long-term evaluation to assess implementation and effectiveness.

Achieving menopause equity requires collaboration across disciplines and institutions, encompassing policymakers, clinicians, researchers, and advocates. When we center the voices and experiences of those navigating menopause, we move closer to a healthcare system that values the health of all individuals across the lifespan. Striving for equity for menopause is not merely an investment in one stage of life; it is an affirmation of dignity, equity, and holistic well-being for generations to come.

## Data Availability

The original contributions presented in the study are included in the article/Supplementary Material, further inquiries can be directed to the corresponding author.
